# Apoptosis of Purified CD4^+^ T Cell Subsets Is Dominated by Cytokine Deprivation and Absence of Other Cells in New Onset Diabetic NOD Mice

**DOI:** 10.1371/journal.pone.0015684

**Published:** 2010-12-31

**Authors:** Ayelet Kaminitz, Enosh M. Askenasy, Isaac Yaniv, Jerry Stein, Nadir Askenasy

**Affiliations:** 1 Frankel Laboratory, Center for Stem Cell Research, Schneider Children's Medical Center of Israel, Petach Tikva, Israel; 2 Bone Marrow Transplant Unit, Schneider Children's Medical Center of Israel, Petach Tikva, Israel; 3 Department of Pediatric Hematology-Oncology, Schneider Children's Medical Center of Israel, Petach Tikva, Israel; 4 Sackler School of Medicine, Tel Aviv University, Tel Aviv, Israel; 5 Soroka Medical School, Ben-Gurion University of the Negev, Beer Sheva, Israel; New York University, United States of America

## Abstract

**Background:**

Regulatory T cells (Treg) play a significant role in immune homeostasis and self-tolerance. Excessive sensitivity of isolated Treg to apoptosis has been demonstrated in NOD mice and humans suffering of type 1 diabetes, suggesting a possible role in the immune dysfunction that underlies autoimmune insulitis. In this study the sensitivity to apoptosis was measured in T cells from new onset diabetic NOD females, comparing purified subsets to mixed cultures.

**Principal Findings:**

Apoptotic cells are short lived in vivo and death occurs primarily during isolation, manipulation and culture. Excessive susceptibility of CD25^+^ T cells to spontaneous apoptosis is characteristic of isolated subsets, however disappears when death is measured in mixed splenocyte cultures. In variance, CD25^−^ T cells display balanced sensitivity to apoptosis under both conditions. The isolation procedure removes soluble factors, IL-2 playing a significant role in sustaining Treg viability. In addition, pro- and anti-apoptotic signals are transduced by cell-to-cell interactions: CD3 and CD28 protect CD25^+^ T cells from apoptosis, and in parallel sensitize naïve effector cells to apoptosis. Treg viability is modulated both by other T cells and other subsets within mixed splenocyte cultures. Variations in sensitivity to apoptosis are often hindered by fast proliferation of viable cells, therefore cycling rates are mandatory to adequate interpretation of cell death assays.

**Conclusions:**

The sensitivity of purified Treg to apoptosis is dominated by cytokine deprivation and absence of cell-to-cell interactions, and deviate significantly from measurements in mixed populations. Balanced sensitivity of naïve/effector and regulatory T cells to apoptosis in NOD mice argues against the concept that differential susceptibility affects disease evolution and progression.

## Introduction

Autoimmune insulitis in NOD mice reflects a condition of disturbed immune homeostasis that involves deregulation of both effector (Teff) and regulatory T cells (Treg). Among multiple aberrant mechanisms detected in NOD mice, emphasis on the sensitivity to apoptosis has been intensely investigated as a possible cause of persistent activity of diabetogenic clones and insufficiency of suppressor mechanisms. Suppressor cells encompass a variety of phenotypes and functions that include both naturally occurring and adaptive regulatory T cells (Treg) [1]–[4]. For pragmatic purposes, high-level expression of CD25 (the α chain of the high affinity IL-2 receptor) is used for purification of naturally occurring Treg [5], which is accompanied by the transcription factor FoxP3 [6]. These subsets are functionally distinct in NOD mice, with CD25^−^ naïve/effector T cells (Teff) transferring the disease efficiently into immunocompromized mice, and CD25^+^ Treg blocking adoptive disease transfer [7]–[9]. The same isolation procedure is widely used to assess various functions of Treg *in vitro*, revealing suppression of Teff proliferation and IL-2 production as major mechanisms of Treg-mediated suppression [10]–[12]. Relative resistance of T cells in general [13], [14] and of effector cells in particular [15], [16], have been attributed a role in disease evolution in NOD mice. These findings question whether differential sensitivities of T cell subsets to apoptosis are involved in dominant effector activity [17].

Increased apoptosis of CD4^+^CD25^+/high^ T cells isolated by FACS sorting and immunomagnetic antibodies from diabetic patients suggests possible involvement in the pathogenesis this autoimmune disorder [18]–[21]. However, despite the apparent increased susceptibility to apoptosis, Treg are found at equal or higher numbers in new onset diabetics and display primarily functional deficits [22]–[25]. As a systemic autoimmune disorder, high levels of circulating Treg reflect a physiological effort to counteract inflammatory reaction, whereas increased susceptibility to apoptosis is an apparent detrimental mechanism. Likewise, Treg levels are steady in NOD females in late stages of destructive insulitis [26], [27], and are found at excessive numbers in mutant mice deficient in Fas-ligand (*gld*) [28]. Although defective negative regulation in *gld* mice might lead to elevated Treg fractions within the generalized lymphoproliferative state, it is unclear why apoptotic cells are collected from wild type mice. Unlike the proliferative anergy displayed by Treg *in vitro* [29], [30], this subset appears to cycle at faster rates *in vivo* [28], [31], a possible cause of increased mortality [17]. Alternatively, it is possible that Treg are particularly susceptible to manipulation during isolation, manipulation and culture and their death is primarily related to the processing procedure. Our prior studies have demonstrated efficient clearance of dead cells from the bone marrow, as determined by intravital microscopy and focused irradiation of labeled cells at the fluorochrome wavelength [32]. We therefore hypothesize that dead cells are scarcely found in tissues under steady state conditions, and death might occur during manipulation.

The sensitivity of purified Treg is modulated by the proliferation rates, cytokines, TCR engagement and the state of activation, and most important by feedback interactions with cytotoxic T cells [17]. Isolation of T cell subsets according to CD25 expression relies on functional assays, where CD25^−^ are largely composed of naïve/effector cells and CD25^+^ correspond to an enriched fraction of naturally occurring Treg expressing FoxP3. For example, CD25^−^ T cells induce insulitis in immunocompromized hosts, whereas CD25^+^ T cells suppress adoptive disease transfer [7]–[9]. In this study we assessed the sensitivity of CD25^−^ and CD25^+^ T cells derived from new onset diabetic mice to spontaneous apoptosis in cultures of purified cells and mixed populations. We found that the isolation procedure is a dominant factor that affects the susceptibility of CD25^+^ T cells. Apoptosis of both effector and regulatory cells is modulated both by IL-2 and cell-to-cell interactions that transduce TCR-associated and costimulatory signals.

## Results

### T cells isolated from lymphoid organs exhibit variations in spontaneous apoptosis

The procedure of immunomagnetic isolation, composed of negative selection of CD4^+^ T cell and positive selection for CD25 within this subset, results in marked variability in lymphocyte viability. These populations are functionally distinct, with CD25^−^ cells adoptively transferring diabetes into 80% of NOD.SCID mice, whereas CD25^+^ T cells lacking diabetogenic activity and repressing disease transfer [9], [26], [27], [29]. A series of 19 isolation procedures showed initial contents of dead cells ranging from 7.6±6.9% (range 0.4%–20%) and 14.3±10.3% (range 0.8%–33%) for CD25^−^ and CD25^+^ subsets respectively. Limiting the use of these samples to 10% dead cells in each individual subset, 13 experiments were used with mean contents of 3.7±2.8% and 6.8±4% non-viable CD25^−^ and CD25^+^ cells, respectively. These values fall within the range previously reported for the frequency of apoptotic cells following cyclophosphamide challenge in vivo [13]. Notably, differential staining showed 7-AAD uptake in 89±5% of the cells which were negative for Annexin-V, indicating non-specific membrane permeabilization as a feature of non-apoptotic death. Since CD25^+^ T cells are more sensitive to death during the preparation procedure (p<0.05 vs CD25^−^), these data present a caution to experiments based on detection of differential sensitivity to apoptosis of purified cells.

To evaluate the precision of measurements of dead cells in fresh samples, NOD mice were infused with 5×10^7^ PKH-labeled dead autologous splenocytes. After 2 hours, 12.8±3.4% of the infused cells were found in the spleens and mesenteric lymph nodes of the NOD recipients. These cells were not detected after 24 hours, demonstrating fast clearance of dead cells entrapped in the lymphoid organs.

### Isolated CD25^+^ T cells are more sensitive to apoptosis than mixed cultures

**Figure 1:**
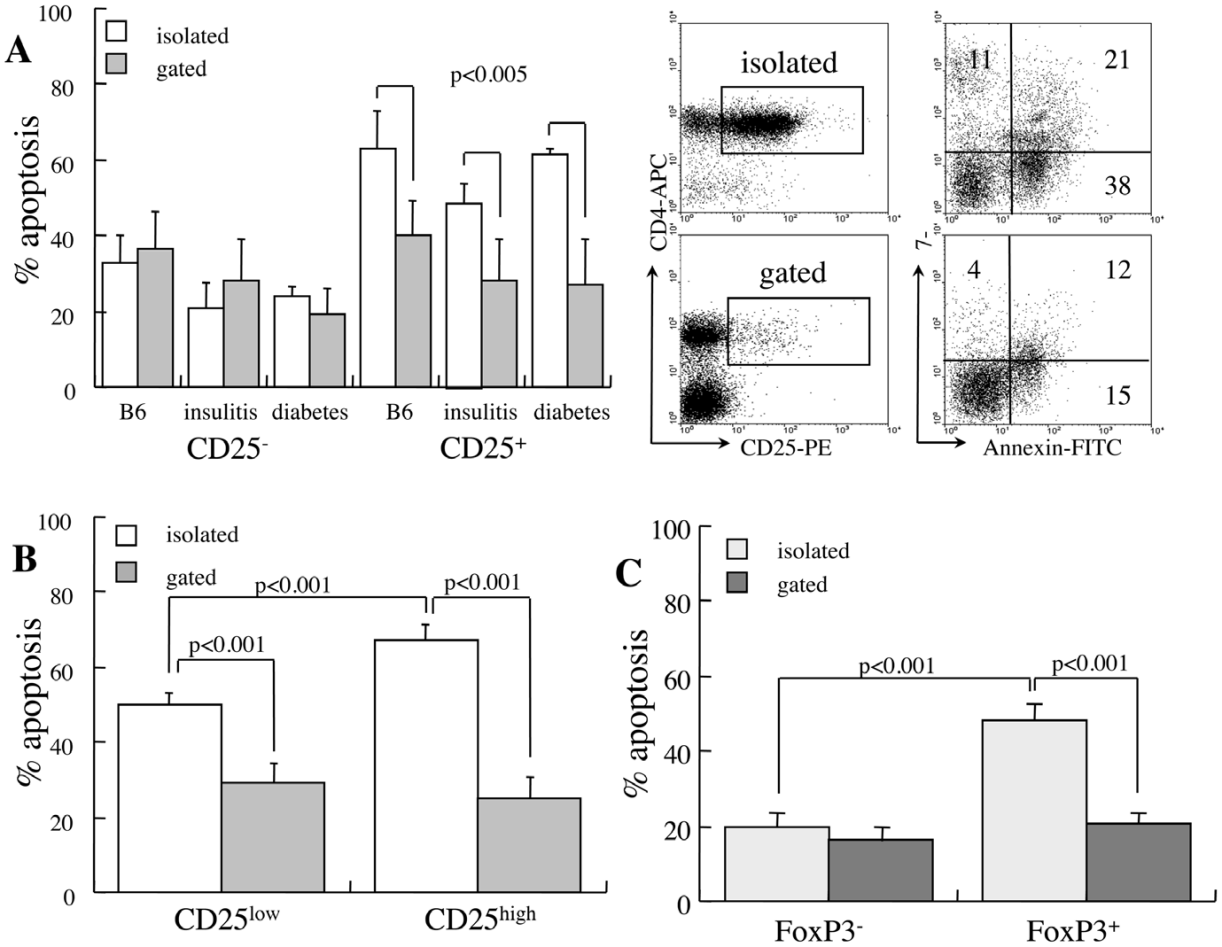
Impact of cell isolation on sensitivity to apoptosis. **A**. Apoptosis of CD4^+^CD25^−^ and CD4^+^CD25^+^ subsets measured after 48 hours in isolated cell suspensions (n = 5) and by gating in mixed cultures (n = 5). Cells were harvested from wild type C57BL/6, prediabetic NOD females aged 14 weeks and new onset diabetic NOD mice. Representative measurements of 7-AAD and Annexin-V incorporation are shown for isolated and mixed populations (gated) from diabetic NOD females. **B**. Differential apoptosis in reference to low and high CD25 expression measured in isolated CD25^+^ subsets (n = 4) and by gating in mixed cultures (n = 5). **C**. Spontaneous apoptosis in reference to FoxP3 expression in the isolated CD25^+^ subset (n = 3) and in mixed cultures (n = 3).

To determine possible variations in susceptibility of CD4^+^ T cell subsets to apoptosis, cells were incubated for 48 hours under conditions of cytokine deprivation. CD4^+^ T cells isolated from wild type (C57BL/6), prediabetic (14 weeks) and diabetic NOD mice display disproportional sensitivity to apoptosis: 21–33% and 48–62% of CD25^−^ and CD25^+^ cells succumb to apoptosis respectively (p<0.01, Figure 1A). Increased apoptosis of CD25^+^ cells isolated from new onset diabetic mice is consistent with the reported sensitivities of CD25^+^ T cells isolated from wild type and *gld* (FasL-defective) mice [28] as well as in humans [18]–[21]. However, measurements of apoptosis performed by gating of CD4^+^ T cell subsets within mixed splenocyte populations show a very different situation (Figure 1A). Increased mortality of CD25^+^ T cells is primarily a result of the isolation procedure (p<0.005), a feature that is much less accentuated in the CD25^−^ subset. Dissociation between CD25^low^ (45%) and CD25^high^ (55%) subsets (Figure 1B) in new onset diabetic NOD females disclosed excessive apoptosis of isolated CD25^high^ cells (p<0.001 vs CD25^low^), suggesting particular dependence on factors released by adjacent cells such as IL-2. Distinct behavior within the CD4^+^ subset was further assessed in reference to FoxP3 expression (Figure 1C), showing increased sensitivity to apoptosis of FoxP3^+^ cells (p<0.001 vs FoxP3^−^) in particular within the isolated CD25^+^ subset (p<0.001 vs gated). These data document increased susceptibility to apoptosis in culture of CD4^+^ T cells displaying a phenotype of naturally occurring Treg derived from wild type and NOD mice.

### Soluble factors and cell-to-cell interactions affect cell viability

**Figure 2:**
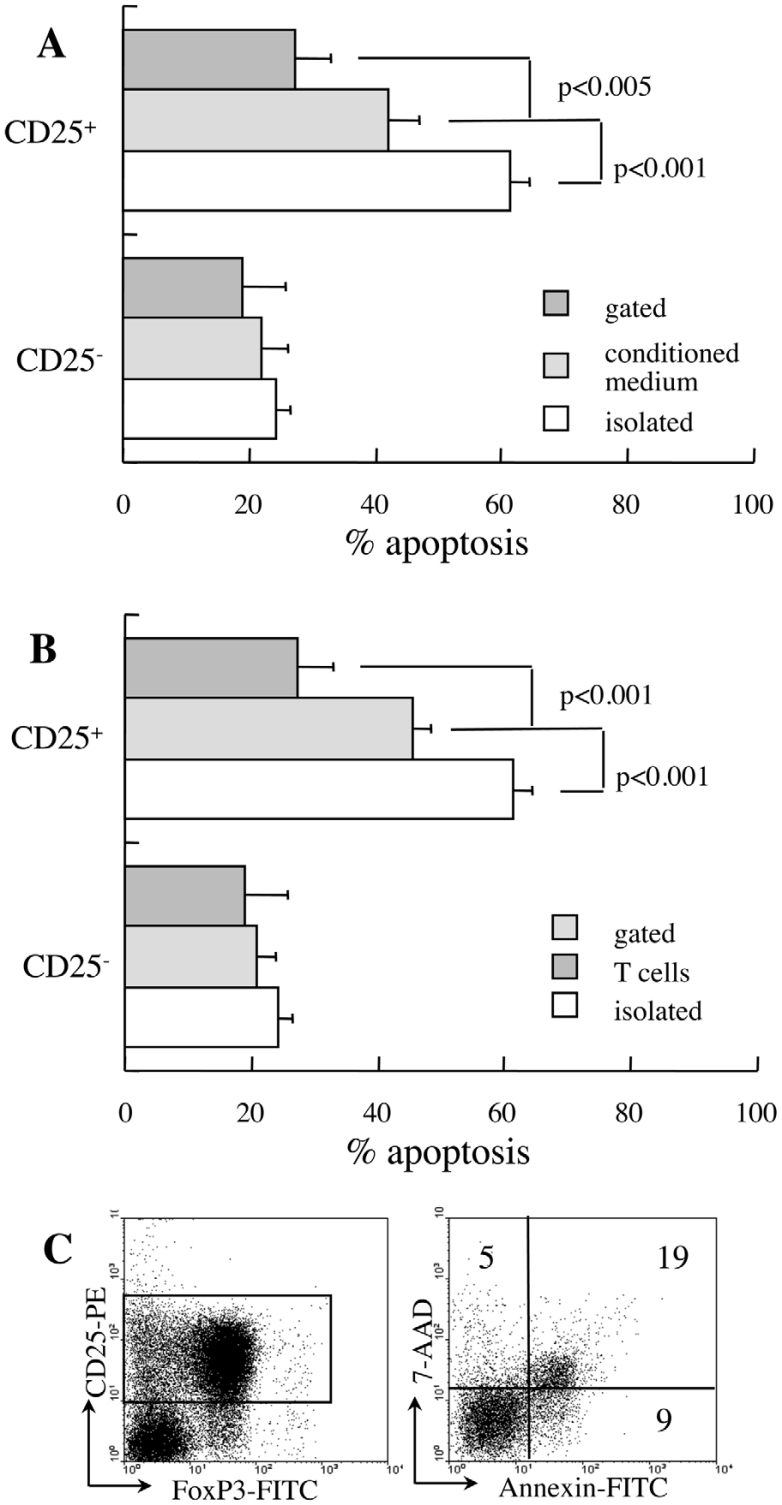
Soluble and cellular factors affecting susceptibility to apoptosis. **A**. CD25^−^ and CD25^+^ T cells isolated from new onset diabetic NOD females were incubated for 48 hours in conditioned medium from CD25^−^ T cells stimulated with surface-bound anti CD3 and anti-CD28 antibodies (n = 3). Data are compared to corresponding measurements of isolated cells and mixed cultures. **B**. Spontaneous apoptosis after 48 hours of culture of isolated CD25^+^ T cells (n = 4) and gated subsets in mixed cultures (n = 5) following B220, GR-1 and MAC-1 depletion (n = 4). **C**. Equal numbers of isolated CD25^−^ and CD25^+^ T cells from diabetic NOD mice were mixed for determination of apoptosis after 48 hours of culture in the CD25+ subset (gate). Data are representative of four independent incubations.

Protection of CD25^+^ T cells from apoptosis by adjacent cells might be mediated by cytokines and cell-to-cell interactions. To determine the role of soluble factors, cells were incubated with conditioned medium of CD25^−^ T cells stimulated in plates containing bound anti-CD3 and CD28 antibodies [33], [34]. Conditioned medium had little influence on apoptotic death of isolated CD25^−^ cells and partially reversed the excessive death of CD25^+^ cells (p<0.001, Figure 2A). Therefore, putative Treg are partially protected from apoptosis in culture by soluble factors released from adjacent populations. To determine the major subsets that affect the sensitivity to apoptosis, B lymphocytes and myeloid cells (GR-1^+^ and MAC-1^+^) were depleted from the mixed cultures (Figure 2B). Since the isolation procedure appears to be a process that sensitizes to apoptosis, we preferred exclusion of cells rather than combining isolated cell subsets. In variance from the small effect of depletion on CD25^−^ cells, exclusion of B lymphocytes and myeloid cells from the cultures partially protected CD25^+^ T cells from apoptosis (p<0.001). Overall, T cells and other constituents of the spleen support the viability of CD25^+^ Treg in culture through release of soluble factors, and possibly through cell-to-cell interactions.

Despite the apparent sensitization to apoptosis during the isolation procedure, the distinct effect on CD4^+^CD25^−^ cells on viability of isolates Treg was performed in co-culture experiments. Incubation of isolated CD4^+^CD25^+^ T cells with an equal number of isolated CD4^+^CD25^−^ was sufficient to reverse susceptibility of the former to spontaneous apoptosis (Figure 2C) and to restore balanced sensitivity to apoptosis characteristic of mixed cultures (Figure 1A).

### IL-2 induces cycling and improves the viability of CD25^+^ T cells

**Figure 3:**
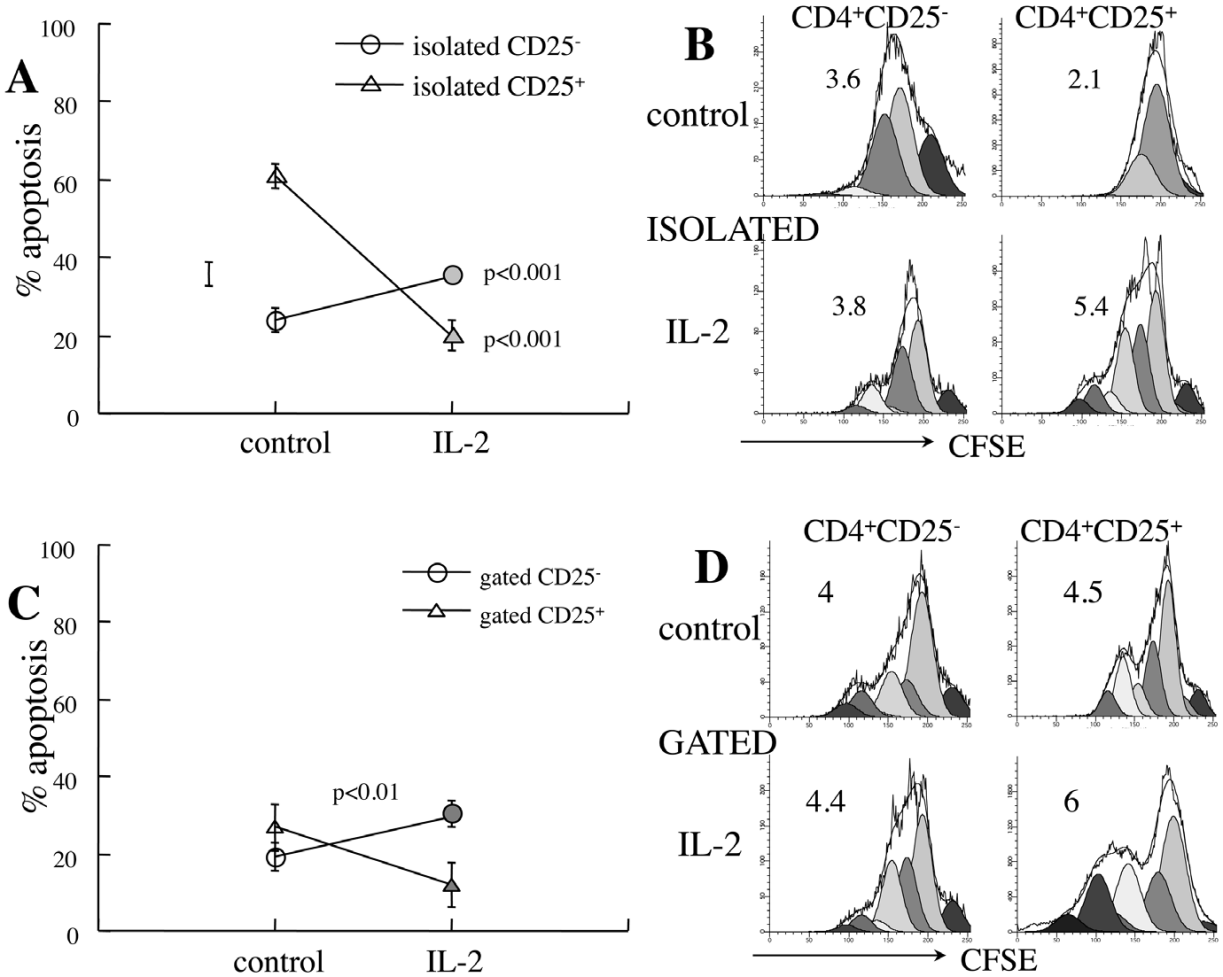
Influence of IL-2 on CD4^+^ T cell apoptosis. **A**. Apoptosis of CD4^+^CD25^−^ (n = 5) and CD4^+^CD25^+^ T cells (n = 4) isolated from new onset diabetic NOD females and incubated for 48 hours with and without 2000 U/ml IL-2. **B**. Demonstrative measurements of proliferation rates of isolated CD25^−^ and CD25^+^ cells as determined from CFSE dilution (representative of 4 independent measurements). **C**. Impact of exogenous IL-2 supplementation on apoptosis measured by gating on CD25^−^ and CD25^+^ subsets in mixed cultures (n = 4). **D**. Demonstrative plots of CFSE dilution in gated CD4^+^ subsets within mixed cultures (representative of 4 measurements).

IL-2 sensitizes T cells to apoptosis [35], however in an apparent paradoxal manner cytokine withdrawal also results in marked increase in susceptibility of Treg cells to apoptosis [36]. The primary cytokine candidate of interaction between Treg and naïve/effector T cells is supplementation of IL-2, an essential cytokine that is avidly consumed and supports expansion, survival and activity of Treg cells [37]. Exogenous IL-2 supplementation to isolated CD25^+^ T cells has a remarkable anti-apoptotic effect on CD25^+^ T cells (p<0.001, Figure 2A) while sensitizing CD25^−^ cells to apoptosis (p<0.001). Decreased fractional death is partially caused by IL-2-mediated stimulation of CD25^+^ T cell proliferation (p<0.05, Figure 3B), diluting the apoptotic cells within a fast cycling viable population. Likewise, IL-2 protects CD25^+^ T cells from apoptosis in mixed cultures (p<0.01, Figure 3C), a phenomenon also associated with faster cycling of this subset (Figure 3D). Therefore, pharmacological concentrations of IL-2 improve the survival of CD25^+^ T cells, both as isolated subsets and within bulk splenocyte populations. Furthermore, the endogenous supply of IL-2 in mixed cultures is insufficient to attain the maximal protective effect over cell viability conferred by this cytokine.

### CD3/CD28 stimulation protects isolated CD25^+^ T cells from apoptosis

**Figure 4:**
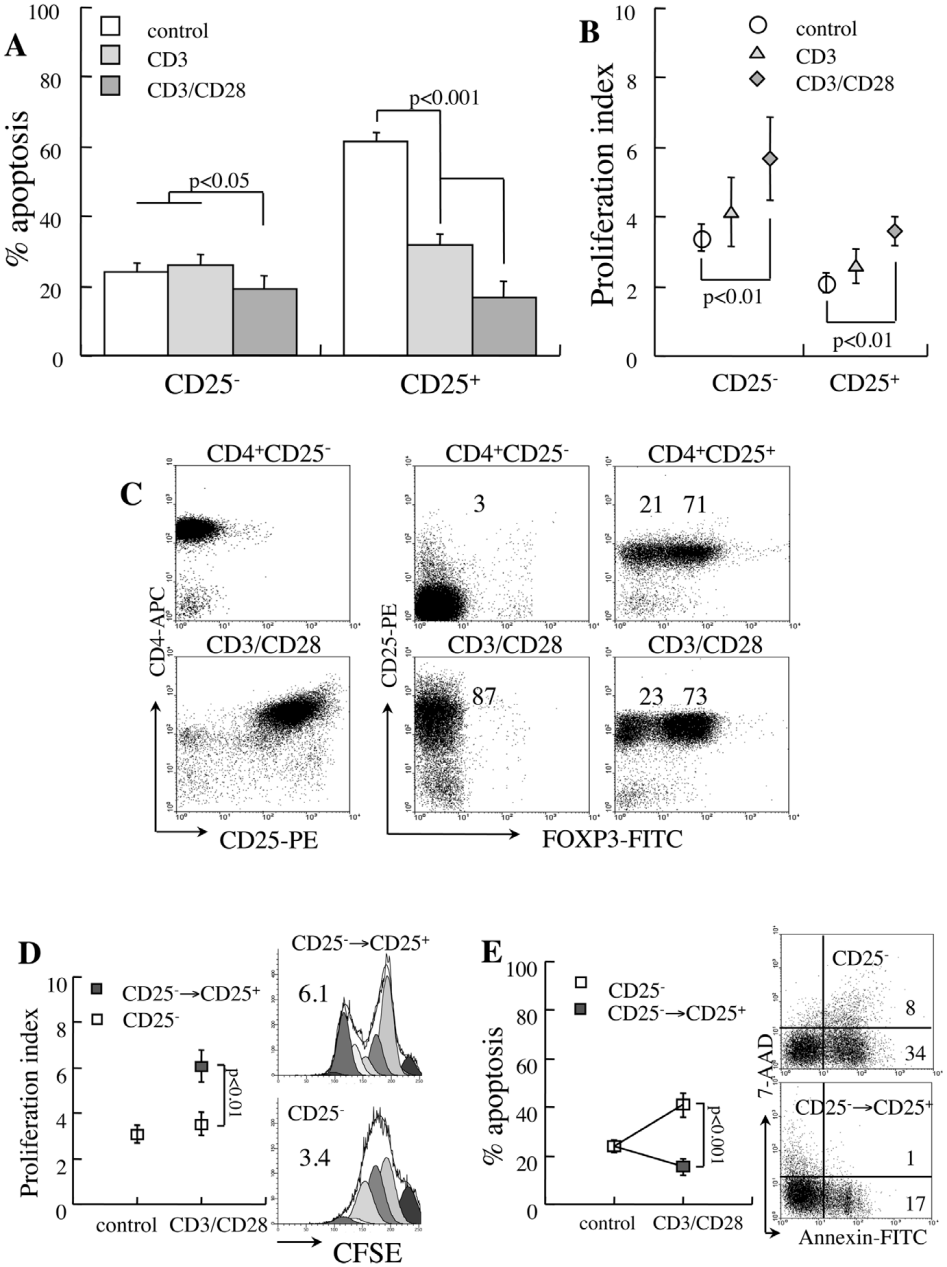
Sensitivity to apoptosis of isolated CD4^+^ T cells under CD3 and CD28 stimulation. CD25^−^ and CD25^+^ T cells isolated from new onset diabetic NOD mice were stimulated with beads conjugated to anti-CD3 and anti-CD28. **A**. Apoptosis during 48 hours of incubation under CD3 (n = 4) and CD3/CD28 stimulation (n = 4). **B**. Proliferation rates determined from CFSE dilution. **C**. Isolated CD25^−^ T cells convert to express CD25 without FoxP3 priming during 48 hours of CD3/CD28 stimulation. Isolated CD25+ T cells sustain Cd25 and FoxP3 expression during Cd3/Cd28 stimulation. **D**. Proliferation rates of cells unresponsive to stimulation (CD25^−^) and the subset that upregulates CD25 expression (CD25^−^→CD25^+^) and representative plots of CFSE dilution. **E**. Fractional apoptosis of responsive and unresponsive CD25^−^ T cells under CD3/CD28 stimulation (n = 4) and representative plots of apoptosis.

Another parameter attenuated by cell isolation is interaction between various cell subsets, including TCR-associated stimulation. Such interactions are stipulated by the observation that the suppressor activity of CD25^+^ T cells depends on cell-to-cell interactions [38], [39] and the sensitivity to apoptosis is reciprocally modulated in coincubations of effector and suppressor cells [33], [34]. Isolated CD25^-^ cells display increased rates of spontaneous apoptosis upon CD3 stimulation with no apparent additional effect of CD28 costimulation (p<0.05, Figure 4A), except stimulation of fast cycling (p<0.01, Figure 4B). Although proliferation sensitizes naïve T lymphocytes to apoptosis, expansion of viable cells dilutes the dead fractions. Both stimulatory signals protect isolated CD25^+^ T cells from spontaneous apoptosis (p<0.001, Figure 4A), with significantly smaller effects on proliferation (Figure 4B). Isolated CD25^+^ T cells maintain fractional expression of the IL-2 receptor and FoxP3 under CD3/CD28 stimulation (Figure 4C). These data are consistent with prior demonstration of protective effects of TCR-associated stimulation over isolated Treg cells and are consistent with successful *ex vivo* expansion of this subset by these stimulatory pathways [40].

Further analysis of isolated CD25^−^ T cells reveals that CD3/CD28 stimulation defines two distinct subsets. A minor fraction of cells remain negative for the IL-2 receptor (Figure 4C), proliferate at slow rates (Figure 4D) and are sensitive to apoptosis (p<0.001, Figure 4E). The major fraction is induced to express CD25 and CD69 without priming FoxP3 expression (Figure 4C), proliferate at faster rates (p<0.01, Figure 4D) and display stable sensitivity to apoptosis (Figure 4E). Although responsive cells (CD25^−^→CD25^+^) display relatively stable levels of fractional apoptosis, fast turnover of viable cells (Figure 4D) dilutes the true numbers of activated apoptotic cells. Taken together, CD3 and CD28 stimulation present dissociated effects on isolated CD4^+^ subsets: increase the sensitivity of isolated CD25^−^ T cells to apoptosis irrespective of the proliferation rates, and improve the viability of isolated CD25^+^ T cells.

### CD28 costimulation sensitizes CD25− T cells to apoptosis in mixed cultures

**Figure 5:**
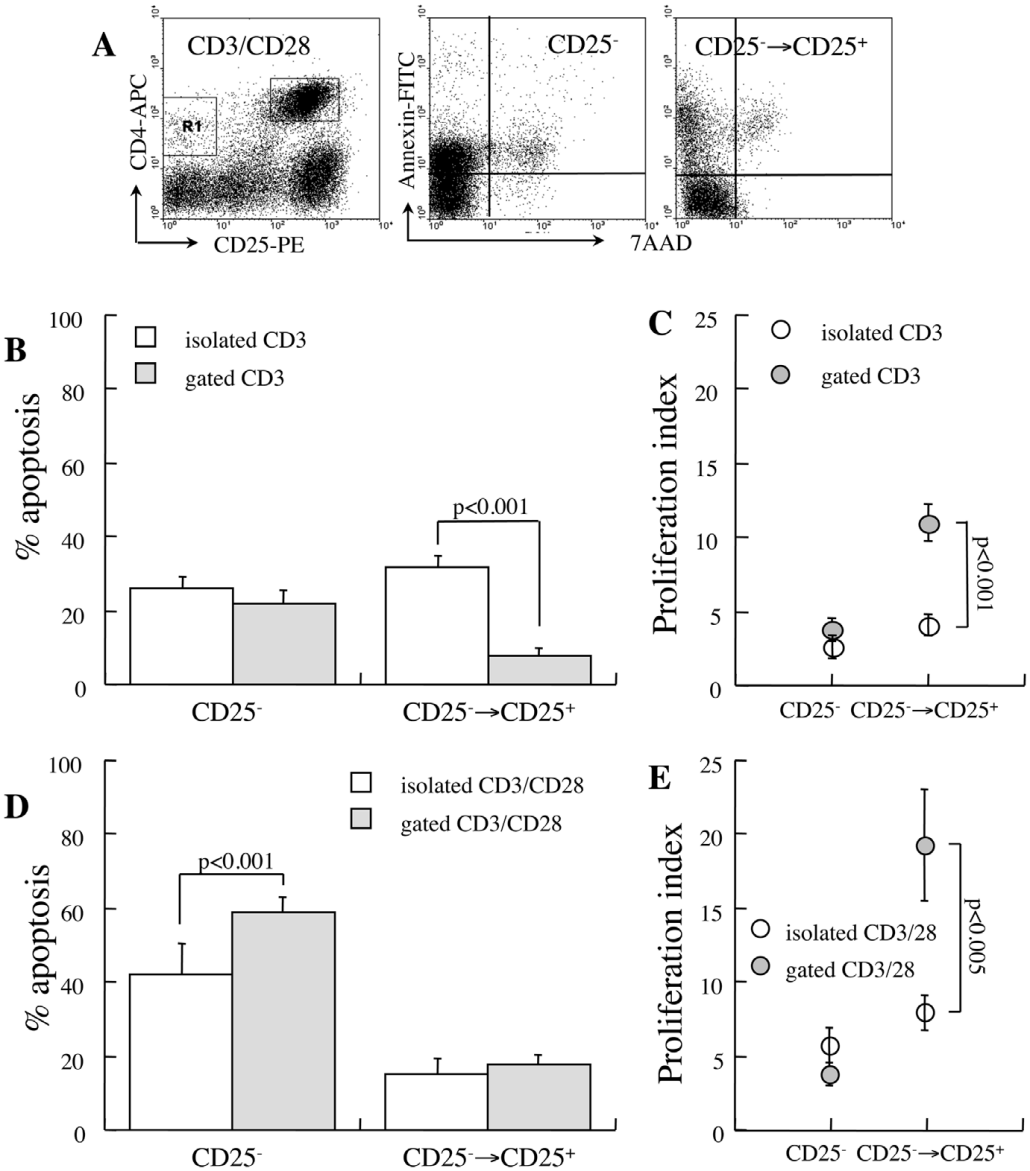
Apoptosis under CD3/CD28 stimulation. **A**. Representative plots demonstrating upregulation of CD25 in response to CD3/CD28 stimulation in mixed splenocyte cultures. **B**. Spontaneous apoptosis in isolated (n = 4) and gated cells (n = 5) in reference to responsiveness (CD25 expression) to CD3-activation, assuming negligible contribution of naturally occurring CD25^+^ T cells in mixed cultures. **C**. Proliferation rates under CD3-activation of responsive (CD25^−^→CD25^+^) and non-responsive (CD25^−^) subsets in isolated CD25^−^ T cells (n = 4) and in mixed cultures (gated, n = 5). **D**. Fractional apoptosis of responsive and non-responsive cells to CD3/CD28 stimulation in mixed cultures (n = 5). **E**. Proliferation rates under CD3/CD28 stimulation.

Measurements of Treg cell apoptosis in CD3-activated mixed cultures are limited by CD25 expression in naïve/effector CD4^+^ T cells as a feature of activation. Evaluation of mixed cultures reveals conversion to express CD25 of 85% of the CD4^+^CD25^−^ T cells (Figure 5A), making the contribution naturally occurring CD25^+^ (7.6±1.3%) and FoxP3^+^ cells (10.9±3%) relatively insignificant. Assuming a negligible contribution of Treg to the fraction of CD3-activated CD4^+^ T cells, the responsive cells display superior survival in mixed cultures (p<0.001 vs isolated cells, Figure 5B). Decreased fractional apoptosis is partially attributed to fast cycling of viable cells (Figure 5C), diluting the apoptotic subset. Superposed CD28 costimulation results in remarkable high death rates on unresponsive cells (Figure 5D), which is even more pronounced in mixed cultures (p<0.001 vs isolated cells). Notably, the proliferative responses to CD3/CD28 stimulation are largely amplified in mixed cultures (p<0.005, Figure 5E), suggesting synergism with other factors secreted by adjacent cells. Overall, these data demonstrate that CD3 stimulation activates naïve/effector CD25^−^ T cells to proliferate at fast rates and display activation markers. Expansion of viable cells is the likely reason for decreased fractional apoptosis of cells responsive to stimulation. Independently, CD28 costimulation sensitizes both unresponsive and responsive subsets to apoptosis, though the latter is hindered by a 4-fold increase in cycling rates.

## Discussion

Measurements performed in isolated cell subsets are easy, precise and allow significant manipulation of multiple experimental variables. Here we provide evidence that assessment of spontaneous apoptosis is dominated by isolation procedures and culture conditions. The first significant observation is wide variability in cell viability in fresh lymphocyte samples, which question whether such measurements can be used to assess differential apoptosis in vivo under various physiological and pathological conditions. Successful harvesting of cells from new onset diabetic mice with less than 1% dead cells, predominant staining with death probes and low specific staining of apoptosis imply that few apoptotic cells are found in lymphoid organs in situ. Although T cells are submitted to negative regulation both in the thymus and the periphery, dead cells are short lived in vivo due to efficient clearance mechanisms [41]–[44]. Our current observations argue that the differences in fresh samples might originate from differential susceptibilities to death under various pathological conditions, which are accentuated by the isolation procedure. Although susceptibility to apoptosis is a feature of cells isolated from wild type and NOD mice, measurements of apoptosis have been used to suggest that increased apoptosis of CD4^+^CD25^high^ T cells, obtained either by immunomagnetic isolation or FACS sorting, is a mechanism involved in the pathogenesis of autoimmune diabetes [18]–[21]. These data have to be carefully evaluated when used to monitor disease evolution [45], as penetration of death probes might be a primary consequence of membrane permeabilization during isolation rather than a physiological feature of the disease [46]. Likewise, it remains to be determined what is the impact of isolation on the suppressive function of apoptosis-sensitive, non-proliferating Treg on effector cells in diabetic patients [47] and NOD mice [34].

Our data confirm the higher sensitivity of isolated CD25^+^ T cells to apoptosis [18]–[21], and reciprocal modulation of this sensitivity between naïve/effector and suppressor T cells [33], [34]. However, all parameters measured in isolated subsets differed from mixed splenocyte cultures, including proliferation rates, apoptotic death, responses to cytokines, TCR-associated stimulation and costimulation. These functions reflect primarily transition to controlled yet arbitrary culture conditions that differ substantially from the physiological environment of these cells. Treg operate within the infiltrates at the site of inflammation [48], [49], under continuously changing cytokine and cellular environments and antigenic stimuli [12], [50]. We have previously postulated that transition to arbitrary in vitro culture conditions causes proliferative anergy [37], whereas Treg cycle at faster rates than naïve/effector T cells in vivo [28], [31], [46]. CD25^−^ naïve/effector cells display a relatively autonomous behavior in respect to the culture conditions [34], however elimination of T cells and other cell subsets increases the susceptibility of isolated Treg to apoptosis, by removing both soluble factors and signals mediated by cell-to-cell interactions. We attempted to reduce differences originating from the isolation procedure per se by exposure of the mixed cultures to the same processing technique without performing the separation. Therefore, starting from fresh samples with similar contents of dead cells, the variations in cell behavior originate primarily from extrinsic factors under our culture conditions. Comparative analysis showed that both pro- and anti-apoptotic factors secreted by other cells within the culture affect the viability of CD25^+^ and CD25^high^FoxP3^+^ T cells.

Exogenous IL-2 supplementation reversed the excessive death of isolated CD25^+^ T cells and restored their proliferative activity, identifying this cytokine as an anti-apoptotic factor secreted by other cell types. IL-2 is produced only by effector and some adaptive Treg, but not by naturally occurring Treg cells [51], [52], and inhibition of its secretion from naïve/cytotoxic T cells is a major mechanism of suppression [29], [38], [39]. The ensuing state of cytokine deprivation induces apoptosis in effector cells [53], and also serves as a regulatory feedback mechanism that downsizes Treg function [37] towards termination of the inflammatory reaction [17]. In addition to an essential role of this cytokine in Treg development and peripheral function [54], [55], the requirement for its exogenous supplementation has been documented in numerous prior in vitro studies of isolated CD25^+^ T cells [38]–[40] and in vivo [56]. Furthermore, exogenous IL-2 supplementation improves Treg viability in mixed cultures, associating expression of the high affinity IL-2 receptor with responsiveness to the cognate ligand. These data also indicate that baseline IL-2 production in mixed cultures is insufficient to exert maximal protection of Treg from apoptosis. Although the isolation procedure also eliminates other cytokines released by adjacent cells that simulate the activity of IL-2 through activation of other receptors [36], its relative deficiency in the inflammatory infiltrates might contribute to dominant diabetogenic cell activity in NOD mice [56], [57].

Partial reversal of Treg hypersensitivity to apoptosis by soluble factors in conditioned medium indicates that cellular interactions affect viability. Similar to the requirement for cell-to-cell interactions to attain Treg-mediated suppression of responder cells [39], [40], reciprocal signals modulate the sensitivity to apoptosis. Both CD3 stimulation and CD28 costimulation converge to impose additive effects on the viability of isolated Treg, which serve as survival factors independent of IL-2 [33], [34], [40]. Antigenic stimulation is generally necessary to initiate Treg proliferation [58], but TCR engagement is not required for functional activation [59], [60]. This dissociation between Treg function and proliferation might be responsible for the relative resistance to apoptosis [29], [30]. At the same time, CD3/CD28 stimulation sensitizes isolated naïve/effector cells to spontaneous death in culture. Robust proliferation induced by CD3/CD28 stimulation hinders the real sensitivities to apoptosis, as dead cells are diluted within fast-expanding viable cells. Therefore, measurements of cell death should include proliferation rates to determine the true meaning of fractional apoptosis determined under various stimulatory conditions. The evident sensitization to apoptosis of isolated CD25^−^ naïve/effector T cells by CD3 ligation was less pronounced in mixed cultures, in particular the responsive subset. In variance, CD28 appears to increase the susceptibility to spontaneous apoptosis despite fast cycling of the stimulated cells, likely through indirect mechanisms that might involve stimulation of cytokine secretion. Nevertheless, these data demonstrate significant variations in susceptibility to apoptosis induced by cell isolation, which attenuates the differential responses to TCR-associated stimulation and costimulation. The presence of adjacent cells involves both pro- and anti-apoptotic signals even under conditions that mediate hyperstimulation of effector cells.

Data presented here emphasize that measurements apoptosis in isolated cell subsets deviate significantly from their behavior in mixed cultures, a feature that reflects a limited capacity of such assays to generate insights into physiological and pathological conditions. Reciprocal influences between effector and regulatory cells are also influenced by other cell types and involve both soluble factors and cell-to-cell interactions. In addition to IL-2 as a dominant cytokine in function of Treg, TCR-associated stimulation and co-stimulation have the capacity to modulate the sensitivity of T cells to apoptosis. Variations in cell death affect the Teff:Treg ratios and consequently impact the severity of inflammatory insulitis in the murine model of type 1 diabetes. Importantly, the data presented here question the validity of the contention that polarized sensitivity to apoptosis measured in isolated subsets underlie the dominance of diabetogenic clones in NOD mice and human diabetics.

## Materials and Methods

### Mice

Mice used in this study were C57BL/6 and non obese diabetic (NOD) purchased from Jackson Laboratories (Bar Harbor, ME). The inbred colonies were housed in a barrier facility. The Institutional Animal Care Committee of the Schneider Medical Center has approved all procedures #022B6229 dated 4.1.2009. Blood glucose was monitored between 9–11 AM in tail blood samples at weekly intervals using a glucometer (Accu-Chek Sensor, Roche Diagnostics, USA). Diabetes was defined as two consecutive blood glucose measurements above 200 mg/dl.

### Cell preparation

Spleens and lymph nodes were harvested and gently minced on a 40 µM nylon mesh in HBSS (Kibbutz Beit Haemek, Israel) to prepare single cell suspensions. Lymphocytes were enriched by centrifugation over 1.5 ml Lympholyte-M (Cedarlane, Ontario, Canada) and T cells were collected after immunomagnetic depletion using antibodies against MAC-1, GR-1. NK1.1 and B220 [61]. All antibodies were obtained from hybridoma cell cultures (ATCC). Antibody-coated cells were washed twice with PBS containing 2% FCS and were incubated with sheep-anti-rat IgG conjugated to M-450 magnetic beads at a ratio of 4–5 beads per cell (Dynal Inc.). Conjugated cells were precipitated by exposure to a magnetic field. The purity of T cell elution was reassessed by flow cytometry using primary labeled monoclonal antibodies against CD4 and CD8. For flow cytometry the red cells were removed by ammonium chloride lysis for 4 minutes at room temperature. The reaction was arrested with excess ice-cold solution and cells were washed.

### Isolation of cells according to CD25 expression

CD25^−^ and CD25^+^ subsets of CD4^+^ T cells are isolated from the spleens and mesenteric lymph nodes using the CD4^+^CD25^+^ Regulatory T cell isolation kit (Miltenyi Biotec, Bergisch-Gladbach, Germany) [62]. In first stage lymphocytes were mixed with a cocktail of biotinylated antibodies against CD8, CD11b, CD45R, CD49b and Ter-119 and incubated with magnetic beads conjugated to anti-biotin antibody. Elution through a column under a magnetic field enriched the unlabeled CD4^+^ T cells. In second stage CD25^+^ was stained with PE-labeled monoclonal antibodies, mixed with anti-PE magnetic microbeads and positively selected by passage through a second column under a magnetic field. Purity was evaluated using FITC-labeled monoclonal antibodies. Immunomagnetic isolation of cells from NOD females yields a CD4^+^CD25^−^ subset contaminated with 0.7±0.4% and 3.6±1.3% CD25^+^ and FoxP3^+^ cells respectively, and a CD4^+^CD25^+^ subset of which 75±4% expressed FoxP3, and are contaminated with 17±8% CD25^−/low^ cells.

### Flow cytometry

Cells were labeled by incubation for 45 minutes at 4°C with the appropriate antibodies conjugated to fluorescein isothyocyanate (FITC), phycoerythrin (PE), allophycocyanin (APC) and peridinin chlorophyll a-protein (PerCP, BD Pharmingen, San Diego, CA): CD4 (clone RM 4–5), CD8 (clone 53-6.7), CD25 (clone PC61.5) [61], [62]. Cells were washed in PBS, incubated for 45 min at 4°C with labeled primary mAb or counterstained with a fluorochrome-labeled secondary mAb. FoxP3 was determined following permeabilization and intracellular staining with a PE-labeled antibody (Foxp3 staining buffer set NRRF-30, eBioscience, San Diego, CA). Antibodies were purchased from BD Pharmingen and eBioscience. Cell death and apoptosis were determined in cells incubated with 5 µg/ml 7-aminoactinomycin-D (7-AAD, Sigma, St. Lois, MO) and Annexin-V (IQ products, Groningen, The Netherlands). Measurements were performed with a Vantage SE flow cytometer (Becton Dickinson, Franklin Lakes, NJ). Positive staining was determined on a log scale, normalized with control cells stained with isotype control antibodies.

### In vitro apoptosis

A concentration of 2×10^6^ cells/ml was prepared in DMEM supplemented with 2 mM L-glutamine, 1 mM sodium pyruvate, 13.6 µM folic acid, 270 µM L-asparagine, 548 µM L-arginine HCL, 10 mM HEPES, 50 µM 2β-Mercaptoethanol, 100 mg/ml streptomycin, 100 U/ml penicillin and 5% heat-inactivated fetal bovine serum (FBS) (MLR medium) [61]–[63]. All ingredients were purchased from Beit Haemek and Sigma (St. Lois, MO). Cells were stimulated by exogenous supplementation of 2000 U/ml IL-2 (Peprotech, London, UK), and beads conjugated to anti-CD3 and anti-CD28 (Invitrogen, Oslo, Norway) at a bead:cell ratio of 1∶1. For determination of apoptosis in reference to FoxP3 expression, cells were first exposed to Annexin-V and subsequently were stained for FoxP3 expression.

### Proliferation assay

Splenocytes and lymphocytes were incubated at room temperature for 7 minutes with 10 µM 5-(and 6-)-carboxyfluorescein diacetate succinimidyl ester (CFSE, Molecular Probes, Carlsbad, CA), and uptake was arrested by addition of 50% FCS. Stained cells were washed with PBS and cultured at 37°C in a humidified 5% CO_2_ atmosphere for 3 days in MLR medium containing 1% heat-inactivated mouse serum [63]. Cells were stimulated with 5 µM concanavalin A (ConA, Sigma) and isolated CD25^+^ T cells were incubated with 20 U/ml IL-2. All proliferation assays were performed in triplicates. Suppression of T cell proliferation was performed in stimulated mixed cultures. Isolated target CD4^+^CD25^−^ T cells were labeled with CFSE and stimulated with an equal number of CD3/CD28 beads. Proliferation was assessed after 48 hours with and without the addition of CD4^+^CD25^+^ T cells at various ratios. CFSE dilution was analyzed in flow cytometry by gating on the live lymphocytes and proliferation was quantified the ModFit software (Verity Software House, Topsham, ME).

### Statistical analysis

Data are presented as means ± standard deviations for each experimental protocol. Results in each experimental group were evaluated for reproducibility by linear regression of duplicate measurements. Differences between the experimental protocols were estimated with a post hoc Scheffe *t*-test and significance was considered at p<0.05.
